# Rural Discontent and Disruptive Politics: A Spatial Justice Perspective

**DOI:** 10.1177/27539687261488823

**Published:** 2026-09-24

**Authors:** Michael Woods

**Affiliations:** 1 Department of Geography and Earth Sciences, 1026 Aberystwyth University, Aberystwyth, UK

**Keywords:** rural discontent, disruptive politics, populism, spatial justice, electoral geography.

## Abstract

This thematic intervention proposes the adoption of a spatial justice framework to examine the drivers of rural support for disruptive political movements and their implications for environmental action. Disruptive parties, especially on the right, have adopted climate-sceptic stances against net zero policies, attributed to the courting of rural support. Yet, the relationship between rural discontent and disruptive politics is not fully understood. The article defines disruptive politics and positions it as part of a wider ‘politics of feeling’. As such, it argues, rural support for disruptive politics cannot be explained by political-economic characteristics or material inequalities, but results from voters interpreting politics through a lens of ‘rural consciousness’ in which rural communities are perceived to be unfairly treated by urban-centred politics and neglected by established parties and institutions. This framing reflects a perception of spatial injustice. The article outlines how spatial justice as a multidimensional concept can provide insights into experiences and perceptions of rural-urban equalities and threats to the ‘right to the rural’, as well as into the consequences of rural populism in compromising the right to the rural of minorities, and provide a normative framework for exploring alternative responses to rural grievances that are more inclusive and empowering.

## Introduction

These are turbulent times for liberal democracies, with established political parties challenged by rising support for disruptive parties, often, though not exclusively, from the radical- or far-right. Among disruptive agendas pursued are attacks on policies aimed at countering climate change, protecting biodiversity, facilitating a green transition, and other environmental goals (Atkins 2022; Deutsch 2021; Allen et al. (2024). For many disruptive parties, their revisionist approach to environmental politics either stems from or is an attempt to exploit rural discontent with controls on agriculture or mining, or with the repurposing of rural land for renewable energy, biodiversity, carbon sequestration or rewilding (Batel, Valquaresma, and Alba 2024; Bujdei-Tebeica 2024; Dechézelles and Scotti 2022; Naumann and Rudolph 2026).

Far-right politicians joined farmers’ protests in Europe in 2023/24 against the impact of green transition policies on agriculture (Bujdei-Tebeica 2024). In the USA, Donald Trump actively has courted rural communities with promises to revive coal-mining and other resource extraction (Billings 2019; Kojola 2019); while part of Bolsonaro's electoral coalition in Brazil were expansionist farmers attracted by pledges to remove constraints on deforestation and roll-back agri-environmental policies (Mendes Motta and Hauber 2023; Pompeia 2024).

Assaults on what had appeared to be a settled environmental consensus are not the only way in which new political actors are disruptive and their amplification of rural environmental grievances is not the only way that rural discontent feeds their support, but they are among the most consequential, presenting a dilemma for mainstream political leaders. Either pro-environmental policies are moderated in response to the electoral threat from disruptive parties, reducing the effectiveness of action to address climate change and biodiversity loss, or policies are pushed through against (rural) opposition, hardening perceptions of political estrangement and further fuelling support for disruptive parties.

In this thematic intervention, I argue that this dilemma cannot be resolved without more fully understanding the drivers of rural support for disruptive politics. This question has generated a plethora of studies over the last decade; however, the findings of these studies lack coherence on both the significance of rurality in shaping electoral geographies and on explanatory factors. Moreover, few studies have focused on individual political outlooks and voting behaviours, or on how these reflect place-based experiences or connect to discursive framings such as populism. I propose that a spatial justice perspective can overcome these limitations. As a multi-dimensional concept, spatial justice provides a framework to connect quantitative and qualitative analyses, engaging with both material and discursive factors. It encompasses analysis of explanations for rural support for disruptive politics, investigation into the consequences of this support, and articulation of spatial justice as a normative idea to explore potential alternative ways of addressing underlying rural grievances. The remainder of the article advances this argument in three parts. First, I define disruptive politics and position them with respect to Anderson and Secor’s (2025) recent thesis of the politics of feeling. Second, I summarise and critique research on rural support for disruptive parties and candidates. Third, I present the spatial justice framework and outline its application to rural discontent and disruptive politics.

## Disruptive Politics and the Politics of Feeling

‘Disruptive politics’ refers to political movements that disrupt settled political cultures and parties in liberal democracies, challenge established leaders and sites of authority, contribute to polarisation, and promote policies that involve significant economic or societal disruption. Disruptive parties, candidates and movements advocate policies that sit outside the settled consensus of acceptable political opinion – from left or right. Examples include economic protectionism, radical economic libertarianism, or anti-capitalism; territorial separatism; reactionary social policies; hardline anti-immigration positions; isolationist or aggressive geopolitical stances; and authoritarian measures. Some disruptive parties promote climate scepticism and opposition to ‘net zero’ or ‘green transition’ policies, but disruptive politics can also, conversely, include radical environmentalism that goes significantly beyond the current mainstream position.

Disruptive political movements also adopt rhetoric and practices that are explicitly polarising or antagonistic. These include, notably, populist discourses that counterpose ‘the people’ to ‘the elite’, rejecting elite institutions and advocating forms of popular sovereignty (Bruhn 2012). Yet not all disruptive politics are populist. Other parties and candidates draw on nationalism, ethnic or religious fundamentalism, radical libertarianism, anarchism, or revolutionary politics. Some disruptive political actors employ language and positions that are openly racist, xenophobic, antisemitic, Islamophobic, misogynistic or homophobic. Some amplify conspiracy theories. Furthermore, some disruptive political movements adopt structures and styles that consciously reject norms, including neophyte leaders, typically drawn from business, media or sport, or leaders who have been ostracised from mainstream politics. In extreme cases, disruptive political parties may have ties to terrorist, paramilitary or criminal organisations.^1^

Disruptive politics can blur boundaries between electoral and extra-parliamentary politics. Social movements such as Occupy, Black Lives Matter and Extinction Rebellion, as well as *Los Indignados* in Spain and the *gilets jaune *in France, have exhibited features of disruptive politics, some morphing into parties, others remaining as vehicles of disillusionment with party politics (farmers’ protests and anti-windfarm demonstrations are not disruptive movements in themselves, primarily focused on policy changes within existing structures, but are prone to capture by disruptive actors). On the far-right, anti-refugee demonstrations form part of an ecosystem of (sometimes violent) protests, online activism and electoral participation. Different manifestations of disruptive politics emerge from variations in political systems, including independent presidential campaigns, insurgent movements within established parties (such as the Tea Party and MAGA movements within the US Republican Party), and referendum campaigns.

Disruptive politics therefore do not form a unified movement. Rather, the term refers to a disparate array of parties, candidates and movements occupying diverse ideological positions, from the far-right, to more moderate right-wing populism and national conservatism, to big-tent populism, to left populism and radical anti-capitalism, to progressive separatism (see supplementary material for a list of identified disruptive parties). To categorise these parties and movements under the same banner is not to suggest any form of moral equivalence. It is, however, to recognise shared outsider status and disruptive intent. From an analytical perspective, starting from the category of ‘disruptive politics’ rather than, say, the more narrowly framed ‘far-right populism’, permits comparison of political trajectories from urban and rural discontent and avoids assuming that rural discontent necessarily leads to right-wing populism.

However, disruptive actors do share discontent with the established order. They exemplify an affective turn that Anderson and Secor identify in contemporary politics, in which ‘politics is motivated and played out through collective political feelings’ (2025, 2). As they expand:

Rather than primarily functioning as coherent programmes of political thought, values, and policy imperatives, today these political forms work by distributing attachment and intensity, operating as collections of rallying points, provocations to outrage, sites of exhilaration or enjoyment, and modes of political performance. (Anderson and Secor 2025, 3)

Although Anderson and Secor present the ‘politics of feeling’ as extending across the political spectrum, describing populism, liberalism and progressivism each as different ‘structures of feeling’ (c.f. Williams 1977), the thesis is especially pertinent to disruptive politics. Support for disruptive political movements is rarely based on a rational assessment of political choices, but on emotional connections and ‘vibes’. The platforms of disruptive parties tend not to be coherent, costed, programmes, but collections of provocative proposals that are meant to instinctively ‘feel right’ to voters. Disruptive movements exploit feelings of fear, insecurity, resentment, betrayal, and alienation; but they also offer supporters more positive feelings of affirmation, belonging, excitement and exhilaration. Disruption itself is an affective act: causing disorientation and confusion for some, but for adherents stimulating satisfaction that *something is being done*.

## Connecting Rural Discontent and Disruptive Politics

Rural support for disruptive political actors is widely alluded to in political, media and academic narratives across several parts of the world (Cramer 2016; de Dominicis, Dijkstra, and Pontarollo 2022; Gimpel and Reeves 2024; Mamonova and Franquesa 2020; Rodden 2019; Thompson 2016). However, evidence from research for this relationship is equivocal. While longitudinal studies have identified a widening rural-urban gap in voting behaviour in the USA (Brown and Mettler 2024; Scala and Johnson 2017), analysis of social surveys in Britain (Claassen, Carmen, and Bankov 2025), Norway (Hesstvedt and Saglie 2025) and globally (Luca et al. 2023) have revealed little difference in political attitudes between rural and urban residents. More geographical analyses have similarly failed to find a consistent, distinctive ‘rural’ influence to explain voting patterns, pointing instead to ‘variegated “place-level despair”’ (Monnat and Brown 2017, 234) or to compositional effects in the rural population (Albrecht 2019, 2022; de Dominicis, Dijkstra, and Pontarollo 2022; Scala, Johnson, and Rogers 2015; Stein, Buck, and Bjørnå 2021). Other studies have argued that geographical peripherality and economic marginalisation are more important than rurality in explaining the tendency for depressed rural regions to back disruptive politics, positioning these as ‘left-behind places’ alongside ex-industrial towns and cities (Broz, Frieden, and Weymouth 2021; de Lange, van der Brug, and Harteveld 2023; Rickardsson 2021).

These findings are salutary corrections to over-simplification of the rural-urban cleavage, but they have methodological limitations. All are based on attempts to quantitatively measure rural impacts on voting behaviour, which encounters the problem that rurality cannot be quantified. Whether using official rural-urban classifications or bespoke models, the analyses rely on proxies for rurality such as population density, remoteness, and agricultural employment. Accordingly, what they show is that there is no direct causality between these proxies – at least in the configuration employed in the model – and voting for disruptive parties and candidates.

An alternative picture is presented by qualitative research that engages with rurality as a social construct, identity, or ‘state of mind’. Here, the emphasis has been less on calculating a rural effect on the size of the vote for disruptive parties, than on understanding the part of rural identity in voter decision-making processes in place.

A series of influential ethnographic studies in the USA by Cramer (2016), Hochschild (2016, 2024), and Wuthnow (2018), showed rural and small-town residents interpreting their experiences of social and economic change through the context of place. A place frame enables individual grievances to be converted into collective concerns and for blame to be assigned to external actors disconnected from the place. This process is not exclusively rural, but Gimpel and Reeves (2024) argue that it is intensified by heightened place attachment in rural communities. For Jacobs and Shea, rural identity is ‘largely informed by notions of linked fate, group consciousness and a sense that the community's well-being is important to a person's own personal success’ (2024, 365), such that rural voters are distinguished as a discrete group in the electorate by concern about their community's future.

It matters that rural communities are not only small and distant from national capitals, but that they are ‘rural’. As residents understand their places to be rural, so perceived economic, social, environmental or political challenges to that place become threats to rural identity. Moreover, rural identity encourages connections to be made between the specific challenges of individual rural places such that a political worldview emerges that is primarily premised on the existence of a rural-urban divide.

Cramer describes this worldview as ‘rural consciousness’, ‘a sense that decision makers routinely ignore rural places and fail to give rural communities their fair share of resources, as well as a sense that rural folks are fundamentally different from urbanites in terms of lifestyles, values, and work ethic’ (2016, 5–6). For Cramer, rural consciousness involves residents interpreting politics through the lens of the places in which they live, overriding other collective identities. In her case study of Wisconsin, Cramer (2016) argues that perceptions that rural tax dollars were being wasted in cities and that rural counties were not getting a fair share of public resources back led not to calls for more government spending, but to support for the radical right-wing Republican Scott Walker and his programme of small government.

Jadhav (2021) similarly outlines a ‘rural articulation praxis’ - grounded in rural tropes of autonomy and self-reliance, scepticism of outsiders, concern for community, and respect for social and economic order – that led voters in the American Mid-West to align their interests with Donald Trump. In Germany, Domann (2024) details a ‘rural rationality’, derived from embedded spatial imaginaries, that fetishises the ‘common sense’ of rural people and normalises far-right politics.

These conceptualisations all assert that rural identity is real, involves interpreting politics through the lens of rural place and community attachment, and has agency to shape political behaviour. Importantly, these dynamics resonate with a politics of feeling. Ruralness is associated with positive feelings of security, familiarity and belonging, but these are perceived to be threatened by outside influences beyond the community's control, stirring up negative emotions. Cramer (2016) emphasises *resentment*, Hochschild (2016) recounts feelings of *betrayal*, *marginalisation*, and *disconnection* among rural voters in Louisiana, while her later work positions a sense of *stolen pride* as an explanation for support for Trump in rural Kentucky (Hochschild 2024).

These emotions are echoed and amplified by disruptive political actors (Domann 2024; Förtner, Belina, and Naumann 2021; Jadhav 2021) and are sometimes embellished by appeals to nostalgia (Kojola 2019) or framings of rural people as guardians of national identity (Breeze 2019; Lambrechts and De Cleen 2025; Limeberry and Fox 2021). Rural discontent is channelled into support for disruptive politics, often aided by the addition of populist rhetoric that reworks the urban-rural divide as tension between honest rural people and an out-of-touch urban elite. It also directs criticism towards the perceived failings of established elite representatives of rural interests, such as farm unions and agrarian and conservative parties (Woods 2015). At the same time, disruptive political actors present themselves as listening to rural communities and embodying rural values. By validating the feelings and instincts of rural voters they offer a more positive emotional experience approaching elation that voters may seek to protect by blocking out any critical perspectives (Hochschild 2016).

Although notions of rural consciousness and rural rationality may imply a singular rural identity, in practice social construction produces multiple imaginaries of ruralness and multiple different interpretations of politics through the lens of rural place, leading to intra-rural differences in the strength, form and direction of support for disruptive politics (Ashwood 2018a; Fourquet 2023; Jacobs and Shea 2024; Rohla et al. 2018). Moreover, differences in political economic structures, including political institutions, cultures and histories, shape variations between regions and between countries in the timing and focus of rural discontent and associated disruptive political movements, the balance between protest and electoral mobilisation, and tendencies towards right-wing populism, left-wing radicalism, separatism or other disruptive platforms (Franquesa 2019; Massetti 2018; Strijker, Voerman, and Terluin 2015; Woods 2015). Indeed, while much of the literature on right-wing disruptive rural politics emanates from Europe and North America, accounts from Africa, Asia and Latin America describe similar dynamics, but with the influence of post-colonialism, peasant movements and local patronage often favouring left-wing disruptive parties (Thompson 2016).

References to rural consciousness or rural rationality can implicitly reproduce pejorative stereotypes. Nagel, for instance, critiques accounts of the populist shift in U.S. politics in which ‘the South is the epicenter of all that is politically amiss’ (2018: 682) but also the ‘inadvertent’ reproduction of southern exceptionalism by critical scholars in representations of southern states as marked by fraught histories of racial exclusion. Hochschild has similarly been critiqued for implicitly reiterating ‘the tired, yet recurring, adage that rural people are … big on muscle, but short on intellect’ (Ashwood 2018a, 738). Rural support for right-wing populist parties and candidates is repeatedly characterised as problematic, paradoxical or irrational (Cramer 2016; Cromby 2019; Hochschild 2016; Jadhav 2021; Maneuvrier-Hervieu, Azzollini, and Jeannet 2025; Petrovici and Poenaru 2025; van der Ploeg 2020; Wallman Lundåsen 2024). In this logic, rural participants in populist and right-wing politics are either part of an oppressor class, to be resisted, or dupes, tricked into backing policies that are contrary to their own interests (see also Ashwood 2018a; Carolan 2020). As Carolan (2020) has observed, this positioning leaves little space for sympathy for the genuine grievances of rural people that fail to align with the expectations of mainstream justice scholars and activists. Accordingly, I argue here that ‘rural consciousness’ and ‘rural rationality’ are not sufficient to understand the connections between rural discontent and disruptive politics and that we need to add a critical but open-minded consideration of rural spatial justice.

## Bringing in Spatial Justice

The concept of spatial justice has twin roots in Harvey’s (1973) discussion of ‘territorial social justice’ and Lefebvre’s (1968) writing on ‘the right to the city’. These perspectives were melded into a more specific notion of urban spatial justice in the work by Pirie (1983), Dikeç (2001) and Soja (2009, 2010), the latter emphasising the duality of spatial justice as process and spatial justice as outcome. Rural applications of spatial justice have been more recent, covering the capacities or capabilities of rural places (Nordberg 2020; Shucksmith, Brooks, and Madanipour 2021), as well as more Lefebvrean ideas of the ‘right to the rural’ (Van Sant and Fairbairn 2026), ‘right to the countryside’ (Barraclough 2013; Naumann and Rudolph 2026), or ‘right to the village’ (Landy and Moreau 2015). This literature has intersected with a longer trajectory of work on rural environmental (in)justice (Ashwood and MacTavish 2016), including inequalities in climate change impacts and in the siting of ‘green’ infrastructure.

In advocating application of spatial justice to disruptive rural politics, I take a slightly broader perspective that encompasses a concern with the capabilities of rural places but also more explicitly engages with concepts of rights, entitlements and deservingness in justice scholarship and ideas of inter-territorial fairness in the distribution of resources and access to power. Spatial justice is here a multilayered framework manifested along five parallel dimensions (see also Woods 2025):

1. Mapping territorial inequalities, reflecting concerns with the spatial distribution of resources.
2. Understanding perceptions of fairness or unfairness in the treatment of places, recognising that inequalities may not only be material but also perceived and include disparities in the distribution of power.
3. Analysis of the spatial production of inequalities, evoking the active reproduction of geographical difference by the state and power elites.
4. The articulation of a ‘right to the rural / the countryside / the village’, and constraints on realising this right, recalling Lefebvre's ‘right to the city’ but also his linked ‘right to difference’.
5. A normative model that employs imaginaries of spatial justice as a basis for emancipatory politics.

The different layers of spatial justice each provide insights into specific aspects of the relationship between rural discontent and disruptive politics. The first layer of territorial inequalities connects with quantitative analyses of spatial patterns of disruptive politics, evidencing political economic factors fuelling discontent, including being economically ‘left behind’ (Broz, Frieden, and Weymouth 2021; Rodríguez-Pose 2018), but also uneven distribution of resources for mitigating climate change impacts, such as flood alleviation schemes. Understanding perceived spatial injustice, in turn, resonates with qualitative research on rural consciousness, which in Cramer’s (2016) formulation includes the perception that rural communities are not given a fair share of resources, as well as contentions that rural interests have been ‘sacrificed’ for green energy or rewilding projects. Campaigns against the perceived concentration of windfarms and solar arrays in rural areas have been co-opted by disruptive parties including the *Rassemblement National* in France, *Lega* in Italy, *Chega!* in Portugal, and Reform UK in Britain (Atkins 2022; Batel, Valquaresma, and Alba 2024; Dechézelles and Scotti 2022). Perceptions of unfair treatment or political neglect (Huijsmans and van der Brug 2025; Hyland, Mascherini, and Lamont 2024; Magalhães and Cancela 2025) may be more powerful than actual material inequality in driving feelings of resentment (Auerbach, Eidheim, and Fimreite 2024; Cramer 2016; Huijsmans 2023) or distrust (Ashwood 2018b; Kirk 2025; Lago 2022) towards urban-based elites. Extending analysis to the spatial production of inequalities permits critical investigation of the veracity of rural grievances as well as consideration of the role of political ideologies such as neoliberalism in creating spatial injustice (Edelman 2021).

The right to the rural/countryside/village, meanwhile, may be read on several levels. Most literally it can document assertations from individuals that their capacity to live or work in a rural community has been undermined by, for example, the loss of mining jobs, closure of public services, or decline in farming – including as a consequence of environmental regulations and repurposing of farmland (Esparcia 2024; Jacobs and Shea 2024; Jadhav 2021; Kojola 2019). Farmers’ protests in the Netherlands against attempts to reduce nitrogen emissions by cutting livestock numbers spawned the agrarian populist *BoerBurgerBeweging* party (BBB), which later turned to the right as an ally of Gert Wilders's PVV (Engelen 2023; van der Ploeg 2020); while Bolsonaro in Brazil sided with farmers whose extractivist practices were threatened by environmental regulations (Pompeia 2024).

A more faithful application of Lefebvre's thesis might focus on the involvement of rural residents in shaping rural places and claims that their autonomy has been eroded by ‘urban interference’, top-down policies or the dominance of urban voter power (Cramer 2016; Huijsmans 2023). Batel, Valquaresma, and Alba (2024), for instance, demonstrate the harnessing of narratives of anti-rural green politics by *Chega!* in Portugal. Engaging the notion of the ‘right to difference’, in turn, highlights how the ascendency of populist and nationalist discourses in rural communities can compromise the right to the rural of individuals who do not fit their imaginaries of rural society – opening avenues for research into the experiences of, for instance, undocumented migrants in Trump's rural heartland, or EU citizens living in Brexit-supporting English villages (Devadoss 2023; Neal et al. 2021; Walter 2021).

Finally, adopting a normative approach to spatial justice offers opportunities both to address the empathy gap in much current research and to proactively explore ways of countering extremism and polarisation. If perceptions of spatial injustice feed rural support for disruptive politics, then it should also be possible ask what ‘spatial justice’ would look like for discontented rural citizens. Such an approach would mean taking rural grievances seriously (Carolan 2020), but without accepting the gloss and pull of disruptive politics. Rather, it could form a foundation for a more emancipatory rural politics that is empowering, inclusive, serious about the climate crisis, and seeks to bridge not widen rural-urban divides (Jadhav 2021; Scoones et al. 2018).

## Concluding Remarks

Spatial justice should be at the core of efforts to understand rural support for disruptive politics. In drawing attention to inequalities in the distribution of resources and services, spatial justice accounts for the role of peripherality, depopulation, and uneven development in seeding rural resentment, but also emphasises the importance of placed-based identities and perceived unfair treatment of places, or spatial injustice, in translating experiences of inequality into political action. While there is no universal rural political economy, place-based resentment becomes rural resentment when politics are viewed through the lens of a rural consciousness that draws on shared tropes and commonly blames rural grievances on neglect and mistreatment by urban-based politicians. This framing in turn resonates with populist rhetoric of the people versus the elite, attracting discontented rural voters to disruptive parties. Legacies of a historical conservative hegemony in many rural regions make right-wing populist and nationalist parties the main beneficiaries; but in much of the Global South, intersections with anti-colonial and peasant politics favour the disruptive left.

Moreover, a Lefebvrean reading of spatial justice highlights disparities in the practice of individuals’ ‘right to the rural’, enabling research to examine how individual fears and experiences meld into collective support for disruptive parties. Employing the language of ‘justice’ is important here, allowing the grievances of rural residents to be recognised without condoning the politics that results. At the same time, a spatial justice approach can also intersect with social justice and racial justice to reveal how rural populism and nationalism compromise the right to the rural of individuals and groups that do not fit their discourses of rurality.

Spatial justice similarly intersects with environmental justice, which returns to the conundrum with which I opened this article: why rural communities that are at heightened risk from environmental change, or which experience environmental injustice in exposure to pollution or noxious land uses, support political actors with anti-environmental agendas. Approaching this question through a spatial justice framework can uncover the dynamics that lead rural communities to feel that they are being sacrificed or treated unfairly by regulations and plans for repurposing rural land originating from urban actors, and how such sentiments build on perceptions of deeper spatial injustices and resulting distrust of urban-centric institutions. Equally, applying spatial justice as a normative concept can provide a way of accommodating rural understandings of what is ‘fair’ into planning for a truly spatially-just transition.

## Supplemental Material

sj-pdf-1-peg-10.1177_27539687261488823 - Supplemental material for Rural Discontent and Disruptive Politics: A Spatial Justice Perspective Supplemental material, sj-pdf-1-peg-10.1177_27539687261488823 for Rural Discontent and Disruptive Politics: A Spatial Justice Perspective by Michael Woods in Progress in Environmental Geography

## Data Availability

All data generated or analysed during this study are included in this published article and its supplementary information files.

## Notes

1. A full definition of disruptive politics can be found at https://ruralspatialjustice.substack.com/p/what-are-disruptive-politics.

## References

[bibr1-27539687261488823] Albrecht D. E. 2019. “The Non-Metro Vote and the Election of Donald Trump.” Journal of Rural Social Sciences 34 (1): 3. https://egrove.olemiss.edu/jrss/vol34/iss1/3 .

[bibr2-27539687261488823] Albrecht D. E. 2022. “Donald Trump and Changing Rural/Urban Voting Patterns.” Journal of Rural Studies 91: 148–156. 10.1016/j.jrurstud.2022.03.009 .

[bibr44-27539687261488823] Allen I. K. Ekberg K. Holgersen S. Malm A. . 2024. Political Ecologies of the Far Right. Manchester: Manchester University Press.

[bibr3-27539687261488823] Anderson B. Secor A. . 2025. The Politics of Feeling: Populism, Progressivism, Liberalism. London: Goldsmiths Press.

[bibr4-27539687261488823] Ashwood L. 2018a. “Rural Conservatism or Anarchism? The Pro-State, Stateless and Anti-State Positions.” Rural Sociology 83: 717–748. 10.1111/ruso.12226 .

[bibr5-27539687261488823] Ashwood L. 2018b. For-Profit Democracy: Why Government in Losing the Trust of Rural America. New Haven: Yale University Press.

[bibr6-27539687261488823] Ashwood L. MacTavish K. . 2016. “Tyranny of the Majority and Rural Environmental Injustice.” Journal of Rural Studies 47: 271–277. 10.1016/j.jrurstud.2016.06.017 .

[bibr7-27539687261488823] Atkins E. 2022. “‘Bigger than Brexit’: Exploring Right-Wing Populism and Net-Zero Policies in the United Kingdom.” Energy Research & Social Science 90: 102681. 10.1016/j.erss.2022.102681 .

[bibr8-27539687261488823] Auerbach K. R. Eidheim M. R. Fimreite A. L. . 2024. “Place-Based Resentment in an Egalitarian Welfare State.” Political Geography 113: 103161. 10.1016/j.polgeo.2024.103161 .

[bibr9-27539687261488823] Barraclough L. 2013. “Is There Also a Right to the Countryside?” Antipode 45 (5): 1047–1046. 10.1111/anti.12040 .

[bibr10-27539687261488823] Batel S. Valquaresma A. Alba M. . 2024. “Rural Communities’ Energy Metabolisms in Portugal: Between Territorial Injustices and Far-Right Populism.” Journal of Rural Studies 11: 103425. 10.1016/j.jrurstud.2024.103425 .

[bibr11-27539687261488823] Billings D. 2019. “Once upon a Time in ‘Trumpalachia’.” In Appalachian Reckoning: A Region Responds to Hillbilly Elegy, edited by Harkins A. McCarroll M. . Morgantown: West Virginia University Press.

[bibr12-27539687261488823] Breeze M. 2019. “Positioning ‘the People’ and Its Enemies: Populism and Nationalism in AfD and UKIP.” Javost – The Public 26 (1): 89–104. 10.1080/13183222.2018.1531339 .

[bibr13-27539687261488823] Brown T. E. Mettler S. . 2024. “Sequential Polarization: The Development of the Rural-Urban Political divide, 1976-2020.” Perspectives on Politics 22 (3): 630–658. 10.1017/S1537592723002918 .

[bibr14-27539687261488823] Broz J. L. Frieden J. Weymouth S. . 2021. “Populism in Place: The Economic Geography of the Globalization Backlash.” International Organization 75: 464–494. 10.1017/S0020818320000314 .

[bibr15-27539687261488823] Bruhn K. 2012. “‘To Hell With Your Corrupt Institutions!’ AMLO and Populism in Mexico.” In Populism in Europe and the Americas, edited by Mudde C. Kaltwasser C. R. . Cambridge: Cambridge University Press.

[bibr16-27539687261488823] Bujdei-Tebeica V. 2024. “Green Policies, Gray Areas: Farmers’ Protests and the Environmental Policy Dilemma in the European Union.” Civil Szemle 21, 31–46. 10.62560/csz.2024.02.03 .

[bibr17-27539687261488823] Carolan M. 2020. “The Rural Problem: Justice in the Countryside.” Rural Sociology 85 (1): 22–56. 10.1111/ruso.12278 .

[bibr18-27539687261488823] Claassen C. Carmen C. Bankov P. . 2025. “Is There a Rural-Urban Political Divide in Britain?” Journal of Elections, Public Opinion and Parties 35 (4): 661–689. 10.1080/17457289.2024.2393587 .

[bibr19-27539687261488823] Cramer K. 2016. The Politics of Resentment. Chicago: University of Chicago Press.

[bibr20-27539687261488823] Cromby J. 2019. “The myths of Brexit.” Journal of Community and Applied Psychology 29 (1): 56–66. 10.1002/casp.2377 .

[bibr21-27539687261488823] Dechézelles S. Scotti I. . 2022. “Wild Wind, Social Storm: ‘Energy Populism’ in Rural Areas? An Exploratory Analysis of France and Italy.” Rural Sociology 87 (S1): 784–813. 10.1111/ruso.12399 .

[bibr22-27539687261488823] de Dominicis L. Dijkstra L. Pontarollo N. . 2022. “Why Are Cities Less Opposed to European Integration Than Rural Areas? Factors Affecting the Eurosceptic Vote by Degree of Urbanization.” Cities 130: 103937. 10.1016/j.cities.2022.103937 .

[bibr23-27539687261488823] de Lange S. van der Brug W. Harteveld E. . 2023. “Regional Resentment in the Netherlands: A Rural or Peripheral Phenomenon?” Regional Studies 57 (3): 403–415. doi:10.1080/00343404.2022.2084527

[bibr24-27539687261488823] Deutsch S. 2021. “Populist Authoritarian Neoliberalism in Brazil: Making Sense of Bolsonaro’s Anti-Environmental Agenda.” Journal of Political Ecology 28: 823–844. 10.2458/jpe.2994 .

[bibr25-27539687261488823] Devadoss C. 2023. “Examining Rust Belt Narratives: Race, Rural Representation, and Everyday Experiences.” Journal of Rural Studies 100: 10310. 10.1016/j.jrurstud.2023.103010 .

[bibr26-27539687261488823] Dikeç M. 2001. “Justice and the Spatial Imagination.” Environment and Planning A 33 (10): 1785–1805. 10.1068/a3467 .

[bibr27-27539687261488823] Domann V. 2024. “Shifting Notions of the Rural: Protests Over Traffic Infrastructure and Far-Right Normalization.” Nordia Geographical Publications 53 (1): 13–38. 10.30671/nordia.122137 .

[bibr28-27539687261488823] Edelman M. 2021. “Hollowed Out Heartland, USA: How Capital Sacrificed Communities and Paved the Way for Authoritarian Populism.” Journal of Rural Studies 82: 505–517. 10.1016/j.jrurstud.2019.10.045 . PMC9758589 36569178

[bibr29-27539687261488823] Engelen E. 2023. Boze burgers en boeren. Rotterdam: Bot uitgevers.

[bibr30-27539687261488823] Esparcia J. 2024. “Rural Depopulation, Civil Society, and its Participation in the Political Arena in Spain: Rise and Fall of ‘Emptied Spain’ as a New Political Actor?” In Win or Lose in Rural Development, edited by Cejudo-García E. Navarro-Valverde F. A. Cañete-Pérez J. A. . Cham: Springer.

[bibr31-27539687261488823] Förtner M. Belina B. Naumann M. . 2021. “The Revenge of the Village? The Geography of Right-Wing Populist Electoral Success, Anti-Politics, and Austerity in Germany.” Environment and Planning C: Politics and Space 39 (3): 574–596. 10.1177/2399654420951803 .

[bibr32-27539687261488823] Fourquet J. 2023. La France D’Après: Tableau politique. Paris: Éditions du Seuil.

[bibr33-27539687261488823] Franquesa J. 2019. “The Vanishing Exception: Republican and Reactionary Specters of Populism in Rural Spain.” Journal of Peasant Studies 46 (3): 537–560. 10.1080/03066150.2019.1578751 .

[bibr34-27539687261488823] Gimpel J. G. Reeves A. . 2024. “The Urban-Rural Divide and Residential Contentment as Antecedents of Political Ideology.” Cities 146: 104720. 10.1016/j.cities.2023.104720 .

[bibr35-27539687261488823] Harvey D. 1973. Social Justice and the City. Baltimore: Johns Hopkins University Press.

[bibr36-27539687261488823] Hesstvedt S. Saglie J. . 2025. “The Urban-Rural Cleavage: Analysing More than 40 Years of Norwegian Survey Data.” Electoral Studies 96: 102954. 10.1016/j.electstud.2025.102954 .

[bibr37-27539687261488823] Hochschild A. R. 2016. Strangers in Their Own Land: Anger and Mourning on the American Right. New York: The New Press.

[bibr38-27539687261488823] Hochschild A. R. 2024. Stolen Pride: Loss, Shame, and the Rise of the Right. New York: The New Press.

[bibr39-27539687261488823] Huijsmans T. 2023. “Place Resentment in ‘The Places that Don’t Matter’: Explaining the Geographic Divide in Populist and Anti-Immigration Attitudes.” Acta Politica 58: 285–305. 10.1057/s41269-022-00244-9 .

[bibr40-27539687261488823] Huijsmans T. van der Brug W. . 2025. “Place-Based Resentment and Party Support in Multiparty Systems. A Study of Three Consecutive Elections in the Netherlands.” Electoral Studies 96: 102963. 10.1016/j.electstud.2025.102963 .

[bibr41-27539687261488823] Hyland M. Mascherini M. Lamont L. . 2024. “Feeling Overlooked: A Rural-Urban Divide in Recognition.” Global Policy 15: 807–822. 10.1111/1758-5899.13454 .

[bibr42-27539687261488823] Jacobs N. F. Shea D. M. . 2024. The Rural Voter: The Politics of Place and the Disuniting of America. New York: Columbia University Press.

[bibr43-27539687261488823] Jadhav A. 2021. “Was It Rural Populism? Returning to the Country, ‘Catching Up’, and Trying to Understand the Trump Vote.” Journal of Rural Studies 82: 553–569. 10.1016/j.jrurstud.2020.10.008 . PMC7580523 33110289

[bibr45-27539687261488823] Kirk J. R. G. 2025. “Landscape of Distrust: Political Trust Across America’s Urban-Rural Divide.” American Politics Research 53 (1): 28–35. 10.1177/1532673X241273220 .

[bibr46-27539687261488823] Kojola E. 2019. “Bring Back The Mines and a Way of Life: Populism and the Politics of Extraction.” Annals of the American Association of Geographers 109 (2): 371–381. 10.1080/24694452.2018.1506695 .

[bibr47-27539687261488823] Lago I. 2022. “Rural decline and satisfaction with democracy.” Acta Politica 57: 753–771. 10.1057/s41269-021-00221-8 .

[bibr48-27539687261488823] Lambrechts G. De Cleen B. . 2025. “Farmers as Symbol of ‘the People’: Nationalism and Populism in Vlaams Belang’s Discourse about Farmers.” Journal of Language and Politics 24 (6): 837–860. 10.1075/jlp.24155.lam .

[bibr49-27539687261488823] Landy F. Moreau S. . 2015. “The Right to the Village.” Justice Spatiale = Spatial Justice 7, https://www.jssj.org/article/le-droit-au-village/?lang=en .

[bibr50-27539687261488823] Lefebvre H. 1968. Le Droit à La Ville. Paris: Anthropos.

[bibr51-27539687261488823] Limeberry V. Fox J. . 2021. “Co-Opting the Rural: Regionalization as Narrative in International Populist Authoritarian Movement Organizing in the United States and France.” Journal of Rural Studies 82: 570–585. 10.1016/j.jrurstud.2020.08.033 .

[bibr52-27539687261488823] Luca D. Terrero-Davila J. Stein J. Lee N. . 2023. “Progressive Cities: Urban-Rural Polarisation of Social Values Economic Development Around the World.” Urban Studies 60 (12): 2329–2350. 10.1177/00420980221148388 .

[bibr53-27539687261488823] Magalhães P. C. Cancela J. . 2025. “Political Neglect and Support for the Radical Right: the Case of Rural Portugal.” Political Geography 116: 103224. 10.1016/j.polgeo.2024.103224 .

[bibr54-27539687261488823] Mamonova N. Franquesa J. . 2020. “Right-Wing Populism in Rural Europe.” Sociologia Ruralis 60: 702–709. 10.1111/soru.12306 .

[bibr55-27539687261488823] Maneuvrier-Hervieu M. Azzollini L. Jeannet A.-M. . 2025. “Cultivating Trust? The Role of European Union Investments in Bridging Rural-Urban Divides.” Electoral Studies 98: 102987. 10.1016/j.electstud.2025.102987 .

[bibr56-27539687261488823] Massetti E. 2018. “Left-Wing Regionalist Populism in the ‘Celtic’ Peripheries: Plaid Cymru and the Scottish National Party’s Anti-Austerity Challenge against the British Elites.” Comparative European Politics 16: 937–953. 10.1057/s41295-018-0136-z .

[bibr57-27539687261488823] Mendes Motta F. Hauber G. . 2023. “Anti-Environmentalism and Proto-Authoritarian Populism in Brazil: Bolsonaro and the Defence of Global Agri-Business.” Environmental Politics 32 (4): 642–662. 10.1080/09644016.2022.2123993 .

[bibr58-27539687261488823] Monnat S. M. Brown D. L. . 2017. “More Than a Rural Revolt: Landscapes of Despair and the 2016 Presidential Election.” Journal of Rural Studies 55: 227–236. 10.1016/j.jrurstud.2017.08.010 . PMC5734668 29269990

[bibr59-27539687261488823] Nagel C. R. 2018. “Southern Exceptionalism and the Perils of Region.” The Professional Geographer 70 (4): 678–686. 10.1080/00330124.2018.1455521.

[bibr60-27539687261488823] Naumann M. Rudolph D. . 2026. “From the ‘Right to Energy’ to the ‘Right to the Countryside’ and Back Again: Contexualizing Rural Energy Transitions.” The Professional Geographer 78: 503–510. 10.1080/00330124.2025.2577916.

[bibr61-27539687261488823] Neal S. Gawlewicz A. Heley J. Jones R. D. . 2021. “Rural Brexit? The Ambivalent Politics of Rural Community, Migration and Dependency.” Journal of Rural Studies 82: 176–183. 10.1016/j.jrurstud.2021.01.017 .

[bibr62-27539687261488823] Nordberg K. 2020. “Spatial Justice and Local Capability in Rural Areas.” Journal of Rural Studies 78: 47–58. 10.1016/j.jrurstud.2020.06.008 .

[bibr63-27539687261488823] Petrovici N. Poenaru F. . 2025. “Revanchism, Misrecognition, and Spatial Configurations: Capital Integration and the Electoral Geography of Romania’s Cancelled 2024 Elections.” Geoforum; Journal of Physical, Human, and Regional Geosciences 166: 104408. 10.1016/j.geoforum.2025.104408 .

[bibr64-27539687261488823] Pirie G. H. 1983. “On Spatial Justice.” Environment and Planning A 15 (4): 465–473. 10.1068/a150465 .

[bibr65-27539687261488823] Pompeia C. 2024. “Agri-bolsonarism: A Movement Led by Agricultural Elites and Far-Right Politicians in Brazil.” Journal of Peasant Studies 51 (7): 1483–1507. 10.1080/03066150.2023.2301440 .

[bibr66-27539687261488823] Rickardsson J. 2021. “The Urban-Rural Divide in Radical Right Populist Support: The Role of Residents’ Characteristics, Urbanization Trends and Public Service Supply.” Annals of Regional Science 67: 211–242. 10.1007/s00168-021-01046-1 .

[bibr67-27539687261488823] Rodden J. A. 2019. Why Cities Lose. New York: Basic Books.

[bibr68-27539687261488823] Rodríguez-Pose A. 2018. “The Revenge of the Places that Don’t Matter (and What to Do About It).” Cambridge Journal of Regions, Economy and Society 11 (1): 189–209. 10.1093/cjres/rsx024 .

[bibr69-27539687261488823] Rohla R. Johnston R. Jones K. Manley D. . 2018. “Spatial Scale and the Geographical Polarization of the American Electorate.” Political Geography 65: 117–122. 10.1016/j.polgeo.2018.05.010 .

[bibr70-27539687261488823] Scala D. J. Johnson K. M. . 2017. “Political Polarization along the Rural-Urban Continuum? The Geography of the Presidential Vote, 2000-2016.” Annals of the Academy of Political and Social Sciences 672: 162–184. 10.1177/0002716217712696 .

[bibr71-27539687261488823] Scala D. J. Johnson K. M. Rogers L. T. . 2015. “Red Rural, Blue Rural? Presidential Voting Patterns in a Changing Rural America.” Political Geography 48: 108–111. doi:10.1016/j.polgeo.2015.02.003

[bibr72-27539687261488823] Scoones I. Edelman M. Borras S. M. Hall R. Jr. Wolford W. White B. . 2018. “Emancipatory Rural Politics: Confronting Authoritarian Populism.” Journal of Peasant Studies 45 (1): 1–20. 10.1080/03066150.2017.1339693 .

[bibr73-27539687261488823] Shucksmith M. Brooks E. Madanipour A. . 2021. “LEADER and Spatial Justice.” Sociologia Ruralis 61 (2): 322–343. 10.1111/soru.12334 .

[bibr74-27539687261488823] Soja E. W. 2009. “The City and Spatial Justice.” Justice Spatiale = Spatial Justice 1, https://www.jssj.org/article/la-ville-et-la-justice-spatiale/?lang=en .

[bibr75-27539687261488823] Soja E. W. 2010. Seeking Spatial Justice. Minneapolis: University of Minnesota Press.

[bibr76-27539687261488823] Stein J. Buck M. Bjørnå H. . 2021. “The Centre-Periphery Dimension and Trust in Politicians: The Case of Norway.” Territory, Politics, Governance 9 (1): 37–55. doi:10.1080/21622671.2019.1624191

[bibr77-27539687261488823] Strijker D. Voerman G. Terluin I. J. . 2015. Rural Protest Groups and Populist Political Parties. Wageningen: Wageningen Academic Publishers.

[bibr78-27539687261488823] Thompson M. R. 2016. “The Moral Economy of Electoralism and the Rise of Populism in the Philippines and Thailand.” Journal of Developing Societies 32 (3): 246–269. 10.1177/0169796X16652028 .

[bibr79-27539687261488823] van der Ploeg J. D. 2020. “Farmers’ Upheaval, Climate Crisis and Populism.” Journal of Peasant Studies 47 (3): 589–605. 10.1080/03066150.2020.1725490 .

[bibr80-27539687261488823] Van Sant L. Fairbairn M. . 2026. “Towards a Right to the Rural?” Dialogues in Human Geography 16: 510–527. 10.1177/20438206251335317.

[bibr81-27539687261488823] Wallman Lundåsen S. 2024. “Rurality and Discontent: Unraveling the Context Effects of Living in Rural Districts in Local Elections on Support for Sweden Democrats.” Journal of Rural Studies 106: 103209. 10.1016/j.jrurstud.2024.103209 .

[bibr82-27539687261488823] Walter B. M. 2021. “Nostalgia and Precarious Placemaking in Southern Poultry Worlds: Immigration, Race and Community Building in Rural Northern Alabama.” Journal of Rural Studies 82: 542–552. 10.1016/j.jrurstud.2019.12.004 .

[bibr83-27539687261488823] Williams R. 1977. Marxism and Literature. Harmondsworth: Penguin.

[bibr84-27539687261488823] Woods M. 2015. “Explaining Rural Protest: A Comparative Analysis.” In Rural Protest Groups and Populist Political Parties, edited by Strijker D. Voerman G. Terluin I. J. . Wageningen: Wageningen Academic Publishers.

[bibr85-27539687261488823] Woods M. 2025. “Rural Recovery or Rural Spatial Justice? Responding to Multiple Crises for the British Countryside.” The Geographical Journal 191 (4): e12541. 10.1111/geoj.12541 .

[bibr86-27539687261488823] Wuthnow R. 2018. The Left Behind: Decline and Rage in Rural America. Princeton: Princeton University Press.

