# Supplementary material for: Rural Discontent and Disruptive Politics: A Spatial Justice Perspective
Source: Prog Environ Geogr. 2026 Sep 24;5(4):468–79. doi: 10.1177/27539687261488823 (PMC13705581; doi:10.1177/27539687261488823)
Supplement: Supplementary file 1 [file sj-pdf-1-peg-10.1177_27539687261488823.pdf]

RURAL-SPATIAL-JUSTICE PROJECT

Disruptive Political Parties

List of disruptive poltical parties identified for 74 liberal democracies applying criteria outlined at:
https://ruralspatialjustice.substack.com/p/what-are-disruptive-politics?r=4ynl43

As at December 2024. For further information contact Michael Woods, m.woods@aber.ac.uk

Country	Party Name (in own language)	English translation	Orientation
* No longer active † Described by at least one source as 'populist'			
**Albania**	*No significant disruptive parties*		
**Argentina**	Partido de los Trabajadores Socialistas (PTS)	Socialist Workers' Party	Left
	Partido Justicialista (PJ) (formerly Union Popular)	Justicalist Party	Left †
	Partido Libertario (PL)	Libertarian Party	Right
**Australia**	Centre Alliance (formerly Nick Xenophon Team)		Centre †
	Family First		Right
	Jacqui Lambie Network		Right †
	Katter's Australian Party (KAP)		Right †
	One Nation		Right †
	United Australia Party (formerly Palmer United Party)		Right †
**Austria**	Bundis Zukunft Österreichs (BZÖ)	Alliance for the Future of Austria	Right †
	Freiheitliche Partei Österreichs (FPÖ)	Freedom Party of Austria	Right †
	JETZT - Liste Pilz	NOW – Pilz List	Mixed †
	Team Karten (TK) (formerly Team Stronach)	Team Carinthia	Right †
**Belgium**	Nieuw-Vlaamse Alliante (NVA)	New Flemish Alliance	Right †
	Parti du Travail de Belgique / Partij van de Arbeid van Belgie (PTB-PVDA)	Workers' Party of Belgium	Left
	Parti Populaire (PP)*	People's Party	Right †
	Vlaams Belang (VB)	Flemish Interest	Right †
	Vlaams Blok (VB)*	Flemish Block	Right †
	Libertair, Direct, Democratisch (LDD) (formerly Lijst Dedecker)	Libertarian, Direct, Democratic	Right †
**Botswana**	Botswana Movement for Democracy (BMD)		Right †
**Brazil**	Partido Liberal (PL) (formerly Partido da Republica)	Liberal Party	Right †
	Partido Novo	New Party	Right
	Partido Renovador Trabalhista Brasileiro (PRTB)	Brazilian Labour Renewal Party	Right †
	Partido Social Liberal (PSL)*	Social Liberal Party	Right †
	Partido Socialismo e Liberdade (PSOL)	Socialism and Liberty Party	Left †
	Patriota (PATRI) (formerly Partido Ecologico Nacional)*		Right
**Bulgaria**	Ataka!	Attack!	Right †
	Bulgarsko Natsionalno Dvizhenie (VRMO)	Bulgarian National Movement	Right †
	Konservativna Bŭlgariya (formerly NFSB)	Conservative Bulgaria	Right †
	Ima Takav Narod (ITN)	There is Such a People	Big Tent †
	Prezaredi Bŭlgariya (PB) (formerly Bŭlgariya Bez Tsenzura)	Reload Bulgaria (formerly Bulgaria without Censorship)	Right †
	Red, Zakonnost I Spravedlivost (RZS)*	Order, Law and Justice	Right †

	Vazrazhdane (V)	Revival	Right †
	Volya (formerly Liberalen alians; Dnes)	Will (formerly Liberal Alliance; Today)	Right †
**Cabo Verde**	*No significant disruptive parties*		
**Canada**	Parti Quebecois (PQ)	Quebec Party	Left
	People's Alliance of New Brunswick (PANB)		Right †
	Quebec Solidaire (QS)	Quebec Solidarity	Left
**Chile**	Convergencia Social (CS)*	Social Convergence	Left
	Partido de la Gente (PDG)	Party of the People	Right †
	Partido Republicano (PLR)	Republican Party	Right †
	Revolucion Democratica*	Democratic Revolution	Left
**Colombia**	Comunes (formerly Fuerza Revolucionario del Comun)	Commons (formerly Common Alternative Revolutionary Force)	Left
**Costa Rica**	Partido Nueva Republica (PNR)	New Republic Party	Right †
	Partido Restauracion Nacional (PRN)	National Restoration Party	Right †
**Croatia**	Domovinski pokret (DP)	Homeland Movement	Right †
	Hrvatska Stranka Prava Dr. Ante Starčević (HSP-AS)*	Croatian Party of Rights – Dr Ante Starčević	Right
	Hrvatski suverenisti (HS)	Croatian Sovereigntists	Right †
	Ključ Hrvatske (KH) (formerly Zivi zid)*	Key of Croatia (formerly Human Shield)	Left †
	Most	Bridge	Right †
	Pravo i Pravda	Law and Justice	Right †
	Radnicka fronta (RF)	Workers' Front	Left
**Cyprus**	Anorthotikó Kómma Ergazómenou Laoú (AKEL)	Progressive Party of Working People	Left †
	Ethniko Laiko Metopo (ELAM)	National Popular Front	Right †
	Evropaiko Komma (EVROKO)*	European Party	Right †
	Neoi Orizontes (NH)*	New Horizons	Right †
	Symmaxia Politon (SYN-SYPOL)*	Citizens' Alliance	Left †
**Czechia**	ANO 2011 [Akce nespokojených občanů]	YES 2011 [Action of Dissatisfied Citizens]	Right † (f Big Tent)
	Česká Pirátská Strana (Pirati)	Czech Pirate Party	Centre
	Přísaha – občanské hnutí	Oath – Civic Movement	Right †
	Strana Prav Obcanu (SPO)*	Party of Civic Rights	Left †
	Svoboda a přímá demokracie (SPD)	Freedom and Direct Democracy	Right †
	Úsvit - Národní Koalice (USVIT) (formerly Úsvit přímé demokracie)*	Dawn – National Coalition (formerly Dawn of Direct Democracy)	Right †
	Věci Veřejné (VV)*	Public Affairs	Big Tent †
**Denmark**	Danmarksdemokraterne (Æ)	Denmark Democrats	Right †
	Dansk Folkeparti (O)	Danish People's Party	Right †
	Enhedslisten – De Rød-Grønn (Ø)	Unity List – the Red-Greens	Left †
	Nye Borgerlige (D)	New Right	Right †
**Dominican Republic**	*No significant disruptive parties*		
**Estonia**	Eesti Konservatiivne Rahvaerakond (EKRE)	Conservative People's Party of Estonia	Right †
**Finland**	Perussuomalaiset (PS)	The Finns Party (formerly the True Finns)	Right †
**France**	Centre National des Independants et Paysans (CNIP)	National Centre of Independents and Peasants	Right †
	Debout la France (DLF)	France Arise	Right †
	Euskal Herria Bai (EH Bai)	Basque Country Yes	Left
	La France Insoumise (LFI)	France Unbowed	Left †
	Ligue du Sud (LS)	League of the South	Right †
	Mouvement pour la France (MPF)*	Movement for France	Right †

	Parti de Gauche (PdG)	Left Party	Left †
	Parti Ouvrier Indépendant (POI)	Independent Workers' Party	Left
	Rassemblement National (RN) (formerly Front National)	National Rally (formerly National Front)	Right †
	Rassemblement pour la France (RPF)*	Rally for France	Right
	Reconquête	Reconquest	Right †
	Picardie debout	Picardy Arise	Left †
**Germany**	Alternative für Deutschland (AfD)	Alternative for Germany	Right †
	Bündnis Sahra Wagenknecht – Für Vernunft und Gerechtigkeit (BSW)	Sahra Wagenknecht Alliance - Reason and Justice	Left †
	Die Linke	The Left	Left †
	Die PARTEI Partei für Arbeit, Rechtsstaat, Tierschutz, Elitenförderung und basisdemokratische Initiative (PARTEI)	Party for Labour, Rule of Law, Animal Protection, Promotion of Elites and Grassroots Democratic Initiative	Satirical
	Nationaldemokratische Partei Deutschlands (NPD)	National Democratic Party of Germany	Right
	Piratenpartei Deutschland	Pirate Party of Germany	Left
**Greece**	Anexartitoi Ellines (ANEL)	Independent Greeks – National Patriotic Alliance	Right †
	Chrysí Avgí (XA)*	Golden Dawn	Right †
	Dimokratikó Patriotikó Kínima – NIKI (NIKI/NIKH)	Democratic Patriotic Movement – Victory	Right
	Ellinikí Lýsi (EL)	Greek Solution	Right †
	Foní Logikís (FL)	Voice of Reason	Right †
	Laïkí Orthódoxi Syllalitírio (LAOS)	Popular Orthodox Rally	Right †
	Métopo Evropaikís Realistikís Anypakoís (MeRA25)	European Realistic Disobedience Front	Left †
	Pléfsi Eleftherías (PE)	Course of Freedom	Left †
	Spartiates (S)	Spartans	Right
	Synaspismós Rizospastikís Aristerás - Proodeftikí Symmachía (SYRIZA)	Coalition of the Radical Left - Progressive Alliance	Left †
**Guyana**	*No significant disruptive parties*		
**Hungary**	Fidesz – Magyar Polgári Szövetség (Fidesz)	Fidesz – Hungarian Civic Alliance	Right †
	Jobbik Magyarországért Mozgalom (Jobbik) (formerly Jobbik – Konzervativok)	Jobbik – Movement for a Better Hungary	Right †
	Magyarország Zöld Pártja (LMP) (formerly Lehet Mas a Politika)	Hungary's Green Party (formerly Politics can be Different)	Left †
	Mi Hazánk Mozgalom (MH)	Our Homeland Movement	Right
**Iceland**	Borgarahreyfingin (B-H)	Civic Movement	Big Tent †
	Miðflokkurinn (M)	Centre Party	Right †
	Piratar (P)	Pirate Party	Left
**India**	Bharatiya Janata Party (BJP)	Indian People's Party	Right †
	Bharat Rashtra Samithi (BRS) (formerly Telangara Rashtra Samithi (TRS))	Indian National Council (formerly Telangara National Council)	Right †
	Communist Party of India (Marxist-Leninist) Liberation		Left
	Naam Tamilar Katchi (NTK)	We Are Tamils	Right
	Samajwadi Party (SP)	Socialist Party	Left †
	Tipra Motha Party (TMP) (formerly Tipra Indigenous Progressive Regional Alliance (TIPRA))		Right

	Viduthalai Chiruthaigal Katchi (VCK)	Liberation Panther Party	Mixed
**Indonesia**	Partai Gerakan Indonesia Raya (Gerinda)	Great Indonesian Movement Party	Right †
	Partai Keadilan Sejahtera (PKS)	Prosperous Justice Party	Right
	Peurte Aceh	Aceh Party	Right
**Ireland**	Aontú	Unity	Right †
	Independent Ireland		Right †
	Neamhspleáigh ar son an Athraithe	Independents for Change	Left †
	Neart le Chéile	Solidarity	Left †
	Pobal Roimh Bhrabús (PBP)	People Before Profit	Left †
	Sinn Fein (SF)	We Ourselves	Left †
**Israel**	HaBayit HaYehudi*	The Jewish Home	Right
	HaYamin HeHadash	New Right	Right
	Likud - National Liberal Movement		Right †
	Mafdal-Religious Zionism (Mafdal-RZ)	National Religious Party – Religious Zionism	Right †
	Noam (aka Lazuz)	Pleasantness	Right
	Otzma Yehudit	Jewish Power (or Jewish Strength)	Right
	Yamina*	Rightwards	Right
	Yisrael Beiteinu	Israel our Home	Right †
**Italy**	Allieanza Nazionale (AN)*	National Alliance	Right †
	Die Freiheitlichen	The Libertarians	Right †
	Forza Italia	Forward Italy	Right †
	Fratelli d'Italia (FdI)	Brothers of Italy	Right †
	Italia dei Valori (IdV)	Italy of Values	Big Tent †
	Lega (formerly Lega Nord)	League (formerly Northern League)	Right †
	Liste JWA	JWA List	Right
	Movimento 5 Stelle (M5S)	Five Star Movement	Left † (f Big Tent)
	Movimento Sociale Fiamma Tricolore (MSTF)	Social Movement Tricolour Flame	Right †
	Partito della Rifondazione Comunista (PRC)	Communist Refoundation Party	Left †
	Radicali Italiani	Italian Radicals	Centre
	Sud chiama Nord (ScN) (formerly De Luca Sindaco d'Italia)	South calls North	Mixed †
	Sinistra Ecologia Liberta (SEL)*	Left Ecology Freedom	Left
	Süd-Tiroler Freiheit	South Tyrolean Freedom	Right
**Jamaica**	*No significant disruptive parties*		
**Japan**	Nippon Ishin no Kai (Ishin)	Japan Innovation Party	Right
	Reiwa Shinsengumi (Reiwa)		Left †
	Sanseito	Do It Yourself	Right †
**Latvia**	Jaunais laiks (JL)*	New Era Party	Right †
	Latvija pirmajā vietā (LPV)	Latvia First	Right †
	Nacionālā Apvienība (NA)	National Alliance	Right †
	Par cilvēcīgu Latviju (PCL) (formerly Kam pieder valsts? (KPV-LV))	For a Humane Latvia (formerly Who Owns the State?)	Right †
**Lesotho**	*No significant disruptive parties*		
**Lithuania**	Darbo Partija	Labour Party	Left †
	Drąsos Kelias*	Way of Courage	Big Tent †
	Lietuvos regionų partija (LRP) (formerly Lietuvos socialdemokratų darbo partija (LSDDP))	Lithuanian Party of Regions (formerly Social Democratic Labour Party of Lithuania)	Left
	Lietuvos valstiečių ir žaliųjų sąjunga (LVZS)	Lithuanian Farmers and Greens Union	Mixed †
	Nemuno aušra (NA)	Dawn of Nemunas	Right †

	Partija tvarka ir teisingumas (TT) (formerly Liberalų Demokratų Partija)*	Order and Justice Party (formerly Liberal Democratic Party)	Right †
**Luxembourg**	Alternativ Demokratesch Reformpartei (ADR)	Alternative Democratic Reform Party	Right †
	Déi Lénk (Lenk)	The Left	Left
	Piratepartei Lëtzebuerg (PPLU)	Pirate Party of Luxembourg	Mixed
**Malaysia**	Parti Islam Se-Malaysia (PAS)	Malaysian Islamic Party	Right †
	Parti Sosialis Malaysia (PSM)	Socialist Party of Malaysia	Left †
**Malta**	*No significant disruptive parties*		
**Mauritius**	Front Solidarite Mauricien (FSM) (formerly Hizbullah)	Mauritian Solidarity Front	Right
**Mexico**	*No significant disruptive parties*		
**Moldova**	Partidul Comunistilor din Republica Moldova (PCRM)	Party of Communists of the Republic of Moldova	Left
	Partidul Socialistilor din Republica Moldova (PSRM)	Party of Socialists of the Republic of Moldova	Left
	Partidul 'SOR' (SOR)*	SOR Party	Right †
**Mongolia**	*No significant disruptive parties*		
**Montenegro**	Bošnjačka stranka	Bosniak Party	Right †
	Demokrstska Crna Gora (DCG)	Democratic Montenegro	Big Tent †
	Demokratska narodna partija (DNP)	Democratic People's Party	Mixed †
	Nova srpska demokratija (NSD)	New Serb Democracy	Right †
	Pokret za promjene (PzP)	Movement for Change	Right †
	Prava Crna Gora (PCG)	True Montenegro	Right †
	Radnička partija (RP)	Workers' Party	Big Tent †
	Ujedinjena Crna Gora (UCG)	United Montenegro	Right
**Namibia**	Christian Democratic Voice (CDV)		Right
	Independent Patriots for Change (IPC)		Big Tent †
	Landless People's Movement (LPM)		Left †
	Namibian Economic Freedom Fighters (NEFF)		Left
**Netherlands**	BoerBurgerBeweging (BBB)	Farmer-Citizen Movement	Right †
	ChristenUnie (CU)	Christian Union	Right †
	Forum voor Democratie (FvD)	Forum for Democracy	Right †
	Juiste Antwoord 2021 (JA21)	Correct Answer 21	Right †
	Leefbaar Nederland*	Liveable Netherlands	Big Tent †
	Lijst Pim Fortuyn (LPF)*	Pim Fortuyn List	Right †
	Partij voor de Dieren (PvdD)	Party for the Animals	Left
	Partij voor de Vrijheid (PVV)	Party for Freedom	Right †
	Socialistische Partij (SP)	Socialist Party	Left †
**New Zealand**	ACT New Zealand (ACT)		Right
	New Zealand First		Big Tent †
	Te Pati Maori	Maori Party	Left
**North Macedonia**	Demokratska Partija na Albancite	Democratic Party of Albanians	Right †
	Levica	The Left	Left †
	Vnatrešna makedonska revolucionerna organizacija – Demokratska partija za makedonsko nacionalno edinstvo (VRMO-DPMNE)	Internal Macedonian Revolutionary Organization – Democratic Party for Macedonian National Unity	Right †
**Norway**	Fremskrittspartiet (FrP)	Progress Party	Right †
	Rødt	Red Party	Left †
	Senterpartiet (SP)	Centre Party	Centre †
**Panama**	*No significant disruptive parties*		

**Philippines**	ACT-CIS Partylist (ACT-CIS)	Anti-Crime and Terrorism Community Involvement and Support	Right †
	Partido Demokratiko Pilipino (PDP)	Philippine Democratic Party	Right † (f Left)
	Partido Federal ng Pilipinas (PFP)	Federal Party of the Philippines	Big Tent †
	Pederalismo ng Dugong Dakilang Samahan (PDDS)	Federalism of the Noble Blooded Association	Right †
**Poland**	AGROunia	Agro Union	Left
	Demokracja Bezosrednia (DB)	Direct Democracy	Big Tent †
	Konfederacja Korony Polskiej (KKP)	Confederation of the Polish Crown	Right
	Kongres Nowej Prawicy (KNP)	Congress of the New Right	Right †
	Kukiz'15		Right †
	Liga Polskich Rodzin (LPR)	League of Polish Families	Right †
	Nowa Nadzieja (NN) (formerly KORWIN)	New Hope	Right †
	Prawo i Sprawiedliwość (PiS)	Law and Justice	Right †
	Ruch Narodowy (RN)	National Movement	Right †
	Samoobrona Rzeczpospolitej Polskiej (SRP)*	Self Defence of the Republic of Poland	Left †
	Suwerenna Polska (SP) (formerly Solidarna Polska)	Sovereign Poland (formerly Solidarity Poland)	Right †
	Unia Polityki Realnej (formerly Platforma Janusza Korwin-Mikke)	Real Politics Union	Right †
**Portugal**	Bloco de Esquerda	Left Block	Left †
	Chega!	Enough!	Right †
	Partido Popular Monárquico (PPM)	People's Monarchist Party	Right
**Romania**	Alianța pentru Unirea Românilor (AUR)	Alliance for the Union of Romanians	Right †
	Partidul Mișcarea Populară (PMP)	People's Movement Party	Right †
	Partidul National Conservator Roman (PNCR)	Romanian National Conservative Party	Right †
	Partidul Neamul Românesc (PNR)	Romanian Nationhood Party	Right †
	Partidul Noua Generație – Creștin Democrat (PNG-CD)	New Generation Party – Christian Democrat	Right †
	Partidul Oamenilor Tineri (POT)	Party of Young People	Right †
	Partidul Poporului – Dan Diaconescu (PP-DD)*	People's Party - Dan Diaconescu	Left †
	Partidul România Mare (PRM)	Greater Romania Party	Right †
	S.O.S. România	S.O.S. Romania	Right †
**Serbia**	Dosta je bilo (DJB)	Enough is Enough	Right †
	Mi - Glas iz Naroda (MI-GIN)	We - Voice from the People	Right †
	Nova Demokratska stranka Srbije (NDSS)	New Democratic Party of Serbia	Right †
	Pokret obnova Kraijevine Srbije (POKS)	Movement for the Restoration of the Kingdom of Serbia	Right
	Pokret Socijalista (PS)	Movement of Socialists	Left
	Ruska stranka (RS)	Russian Party	Right
	Srpska napredna stranka (SNS)	Serbian Progressive Party	Big Tent †
	Srpska narodna partija (SNP)	Serbian People's Party	Right †
	Srpski patriotski savez (SPAS)*	Serbian Patriotic Alliance	Right †
	Srpski pokret Dveri (Dveri)	Serbian Movement 'Doors'	Right †
	Srpski pokret obnove (SPO)	Serbian Renewal Movement	Right
	Srpska radikaina stranka	Serbian Radical Party	Right †
	Srpska stranka Zavetnici	Serbian Party of Oathkeepers	Right †
	Zdrava Srbija (ZS)	Healthy Serbia	Right †
**Singapore**	*No significant disruptive parties*		
**Slovakia**	Komunisticka strana Slovenska (KSS)	Communist Party of Slovakia	Left †

	Kotlebovci – Ľudová strana Naše Slovensko (L'SNS) (formerly Slovenska Pospoliost)	People's Party Our Slovakia (formerly Slovak Togetherness)	Right †
	Sloboda a solidarita (Saska)	Freedom and Solidarity	Right
	Kresťanská únia (KU)	Christian Union	Right
	Slovenská Národná Strana (SNS)	Slovak National Party	Right †
	Slovensko (formerly Obycajni ludia a nezavisie osobnosti (OL'aNO))	Slovakia (formerly Ordinary People and Independent Candidates	Right †
	Slovenský PATRIOT	Slovak Patriot	Right
	Smer – Sociálna Demokracia (Smer)	Direction - Social Democracy	Left †
	Sme Rodina (SR) (formerly Nas Kraj)	We are Family (formerly Our Land)	Right †
	Vlast (formerly Dnes; Obcianska strana Dnes; Strana obcianskej lavice)*	Homeland (formerly Today; Civic Party Today; Civic Left Party)	Right †
	Život – Národná strana (formerly KDZP)	Life – National Party	Right
**Slovenia**	Levica	The Left	Left †
	Lista Marjana Sarca (LMS)*	Marjana Sarca List	Big Tent †
	Nova Slovenija – Krščanski Demokrati (N.Si)	New Slovenia – Christian Democrats	Right †
	Slovenska Demokratska Stranka (SDS)	Slovenian Democratic Party	Right †
	Slovenska Nacionalna Stranka (SNS)	Slovenian National Party	Right †
**South Africa**	African Transformation Movement (ATM)		Right †
	Economic Freedom Fighters (EFF)		Left †
	National Coloured Congress (NCC)		Mixed
	Pan-Africanist Congress of Azania (PAC)		Left
	Patriotic Alliance (PA)		Right †
	uMkhonto weSizwe (MK)	Spear of the Nation	Left †
	United Africans Transformation (UAT)		Left
	Vryheidsfront Plus (VF+)	Freedom Front Plus	Right †
**South Korea**	Tonghap Jinbo-dang (UPP)*	United Progressive Party	Left
**Spain**	Adelante Andalucia	Forward Andalusia	Left †
	Anticapitalistas	Anti-capitalists	Left
	Candidatura d'Unitat Popular (CUP)	Popular Unity Candidacy	Left
	Convergencia Democratica de Catalynya (CDC)*	Democratic Convergence of Catalonia	Right
	Esquerra Republicana de Catalunya (ERC)	Republican Left of Catalonia	Left
	Euskal Herria Bildu (EH Bildu)	Basque Country Unite	Left
	Euskal Herrialdeetako Alderdi Komunista (EHAK)*	Communist Party of the Basque Homelands	Left
	Geroa Bai (formerly Nafarroa Bai)	Yes to the Future (formerly Navarra Yes)	Left
	Junts per Catalunya (Junts)	Together for Catalonia	Right †
	Parttit Democrata Europeu Catala (PDeCAT) (formerly Partit Democrata Catala)*	Catalan European Democratic Party (formerly Catalan Democratic Party)	Right
	Podemos	We Can	Left †
	Se Acabo La Fiesta (SALF)	The Party is Over	Right †
	Sumar	United	Left †
	Union do Povo Galego (UPG)	Galician People's Union	Left
	Vox	Voice	Right †

**Sri Lanka**	Janatha Vimukthi Peramuna (JVP)	People’s Liberation Front	Left
	Jathika Hela Urumaya (JHU)	National Heritage Party	Right
	Jathika Jana Balawegaya (JJB)	National People’s Power	Left †
**Suriname**	*No significant disruptive parties*		
**Sweden**	Junilistan*	June List	Mixed
	Piratpartiet	Pirate Party	Left
	Sverigedemokraterna (SD)	Sweden Democrats	Right †
**Switzerland**	Mouvement Citoyens Genevois (MCG)	Geneva Citizens’ Movement	Right †
	Schweizerische Volkspartei / Union démocratique du centre (SVP/UDC)	Swiss People’s Party	Right †
**Taiwan**	Taiwan Jijin (TSP)	Taiwan Statebuilding Party	Left
	Taiwan Solidarity Union (TSU)		Right †
**Thailand**	Palang Pracharath Party (PPRP)	People's State Power Party	Right †
	People's Power Party (PPP)*		Big Tent †
	Pheu Thai Party (PTP)	For Thais Party	Big Tent †
	Thai Rak Thai Party (TRT)*	Thais Love Thais Party	Big Tent †
	United Thai Nation Party (UTN)		Right
**Timor-Leste**	*No significant disruptive parties*		
**Trinidad & Tobago**	*No significant disruptive parties*		
**Türkiye**	Adalet ve Kalkinma Partisi (AK)	Justice and Development Party	Right †
	Halklariin Demokratik Partisi (HDP)	Peoples’ Democratic Party	Left †
	Huda Dava Partisi (HUDA PAR)	Free Cause Party	Right
	Iyi Parti (IYI)	Good Party	Right †
	Milliyetci Hareket Partisi (MHP)	Nationalist Movement Party	Right †
	Türkiye Isci Partisi (TIP)	Workers’ Party of Turkey	Left †
**United Kingdom**	British National Party (BNP)		Right †
	Democratic Unionist Party (DUP)		Right †
	Plaid Cymru	Party of Wales	Left
	Pobal Roimh Bhrabús (PBP)	People Before Profit	Left †
	Reform UK (RUK) (formerly Brexit Party)		Right †
	Respect*		Left †
	Scottish National Party (SNP)		Left
	Sinn Fein (SF)	We Ourselves	Left †
	Traditional Unionist Voice (TUV)		Right
	United Kingdom Independence Party (UKIP)		Right †
	Workers’ Party of Britain (WPB)		Left †
**Uruguay**	Cabildo Abierto (CA)	Open Cabildo	Right †
	Identidad Soberana (IS)	Sovereign Identity	Big Tent †
	Unidad Popular (UP)	Popular Unity	Left
**USA**	Republican Party [from 2020]		Right †
